# Tumour immune microenvironment prognostic factors in locally advanced rectal cancer, a systematic review

**DOI:** 10.3389/fonc.2025.1688696

**Published:** 2025-12-19

**Authors:** Alasdair Ball, Rebecca Lefroy, Malcolm Price, David McArthur, Andrew Beggs

**Affiliations:** 1 Institute for Biomedical Research, Department of Cancer and Genomic Sciences, University of Birmingham, Birmingham, United Kingdom; 2 Department of Surgery, University Hospitals Birmingham NHS Foundation Trust, Birmingham, United Kingdom; 3 Department of Surgery, The Shrewsbury and Telford Hospital NHS Trust, Shrewsbury, United Kingdom; 4 Institute of Applied Health Research, Department of Applied Health Sciences, University of Birmingham, Birmingham, United Kingdom

**Keywords:** rectal cancer, prognosis, lymphocyte, macrophage, immune system

## Abstract

**Introduction:**

Understanding factors influencing individual survival outcomes following surgical resection of locally advanced (LARC) rectal cancer remains challenging. Novel biomarkers could show emerging promise in this setting. This study aimed to systematically review the literature on immune prognostic factors in LARC.

**Methods:**

The review protocol was preregistered on the PROSPERO database (CRD42023460541). Included studies were required to report overall survival and at least one immune prognostic factor for at least ten patients with LARC. Final searches of MEDLINE, EMBASE and Central were concluded on 8th September 2023. The risk of bias was assessed using the QUIPS tool.

**Results:**

22 retrospective cohort studies involving 2,622 LARC patients were included in the review. We did not find any published data on immune prognostic factors in locally recurrent rectal cancer. Due to inconsistency of immune prognostic factor definitions and measurement methods, meta-analysis would not be meaningful. Instead, the results are presented descriptively. Risk of bias was concentrated in the participation, attrition, and confounding domains. Greater cytotoxic cell infiltration was associated with improved overall survival. There was inconsistent evidence of an association of PD-L1 expression and survival. M2 macrophage infiltration and homozygous germline FPR1 loss-of-function were associated with worse survival.

**Discussion:**

These findings support a role for both innate and acquired immune systems in mediating outcomes following surgery for LARC and suggest that further work into immunomodulation may show promise in improving LARC treatment.

**Systematic Review Registration:**

https://www.crd.york.ac.uk/PROSPERO/, identifier [CRD42023460541].

## 1 Introduction

Colorectal cancer is the third most commonly diagnosed cancer worldwide and is responsible for the second greatest number of annual cancer deaths (1, 2). One third of colorectal cancers occur in the rectum or rectosigmoid junction (3, 4), and approximately 10% of these are locally advanced at presentation. Following surgical resection, approximately 10% of locally advanced rectal cancers will recur in the pelvis (5). Where locally advanced (LARC) and locally recurrent (LRRC) rectal cancer is not associated with metastatic disease, multimodal treatment with curative intent remains possible.

Surgery for LARC or LRRC is highly morbid and remains a challenge due to: the difficulties of operating in the confines of the pelvis; fibrosis of surgical planes due to neoadjuvant radiotherapy; and the possible involvement of adjacent organs or structures. Median survival after pelvic exenteration for LARC or LRRC is approximately 3 years (6, 7) and major complications (Clavien-Dindo grade 3a or greater) occur in one third of patients (6–8). One in ten patients will require an unplanned perioperative return to theatre following surgery (6, 7, 9). Quality of life is severely negatively affected in the immediate post-operative period, and gradually recovers over the following 24 months (10). Therefore, judicious case selection is important. Validated prognostic markers could be used to counsel patients prior to surgery, inform case selection, and plan enhanced surveillance in patients at highest risk.

Following the discovery that lymphocytic infiltration in the colorectal cancer microenvironment is associated with improved survival (11), the tumour immune environment has been increasingly examined for prognostic features. However, a previous systematic review of studies, including patients undergoing exenterative surgery for LARC (12), identified a lack of evidence on whether immune features are associated with prognosis in this setting. Adopting more inclusive definitions of LARC and LRRC may identify specific immune prognostic factors that warrant further investigation in the setting of exenterative surgery. We aim to systematically review the literature on tumour immune microenvironment prognostic factors in LARC and LRRC.

## 2 Methods

### 2.1 Registration

The review protocol was pre-registered on the PROSPERO database (CRD42023460541) and adheres to the PRISMA guidelines (13).

### 2.2 Inclusion and exclusion criteria

Randomised controlled trials, cohort studies, and case series with greater than ten patients, including patients with LARC or LRRC, were considered for inclusion. Pragmatic definitions of LARC and LRRC were used: any study reporting inclusion of LARC or LRRC patients was considered for inclusion, regardless of whether or not LARC and LRRC were further defined. Included studies were required to report how at least one prognostic factor relating to the tumour immune microenvironment was associated with overall survival. Studies where the outcomes were not clearly reported or contained no extractable data were excluded.

Where studies presented Kaplan-Meier survival curves without hazard ratios available, figures were examined for the possibility of data extraction using the method of Guyot (14). As estimation of the hazard ratio with incomplete data is likely to be inaccurate (14) high-resolution images of the survival curve and availability of a risk table were required, and studies not meeting this requirement were excluded.

Studies where outcomes for other pathologies were not reported separately were excluded. Abstracts presented at conference, letters and editorials, news articles, commentaries, and review articles were also excluded.

### 2.3 Search strategy

The MEDLINE, EMBASE and Cochrane Central Register of Controlled Trials (CENTRAL) were searched using terms relating to locally advanced or locally recurrent rectal cancer, the immune system and tumour immune microenvironment, and survival outcomes. The search strategies (presented fully in **Supplementary Tables 1**-**3**) were adapted from previous systematic reviews on locally advanced rectal cancer (12) and colorectal peritoneal metastases (15). Searches were restricted to studies published since January 2003 in the English language. Reference lists of included articles were screened to identify additional studies of interest.

### 2.4 Abstract and full-text screening

The titles and abstracts of studies identified by the search strategy were screened by two independent reviewers, and discrepancies were resolved following discussion and mutual agreement. Full texts were screened by two independent reviewers, and eligibility for inclusion was judged against the pre-defined inclusion criteria. Discrepancies at this stage were resolved by agreement with a consultant colorectal surgeon.

### 2.5 Data collection

Data were extracted onto a pre-designed data collection tool. The primary outcome measure was overall survival. Study metadata, and demographic and clinical data were extracted. As included studies were permitted to define “locally advanced” and “locally recurrent” arbitrarily, data on the definitions of LARC and LRRC used were also captured. Many studies presented unadjusted hazard ratios only, and others adjusted for differing confounding factors, unadjusted hazard ratios were preferentially extracted where possible.

### 2.6 Assessment of risk of bias

The risk of bias in individual studies was assessed by the Quality in Prognosis Studies (QUIPS) tool (16) which includes 6 domains: participation; attrition; prognostic factor measurement; outcome measurement; confounding; and analysis and reporting.

### 2.7 Data analysis

A significant degree of clinical and measurement heterogeneity amongst the studies was anticipated. Where cohorts and prognostic factor measurement methods are homogenous, meta-analysis could be meaningful, and it was planned to be undertaken using a random-effects model with 95% prediction intervals. Subgroup analysis with a pre-specified subgroup of patients with T4 disease was planned. Where heterogeneity was identified, meta-analysis was not performed.

## 3 Results

### 3.1 Searches and article screening

**Figure 1:**
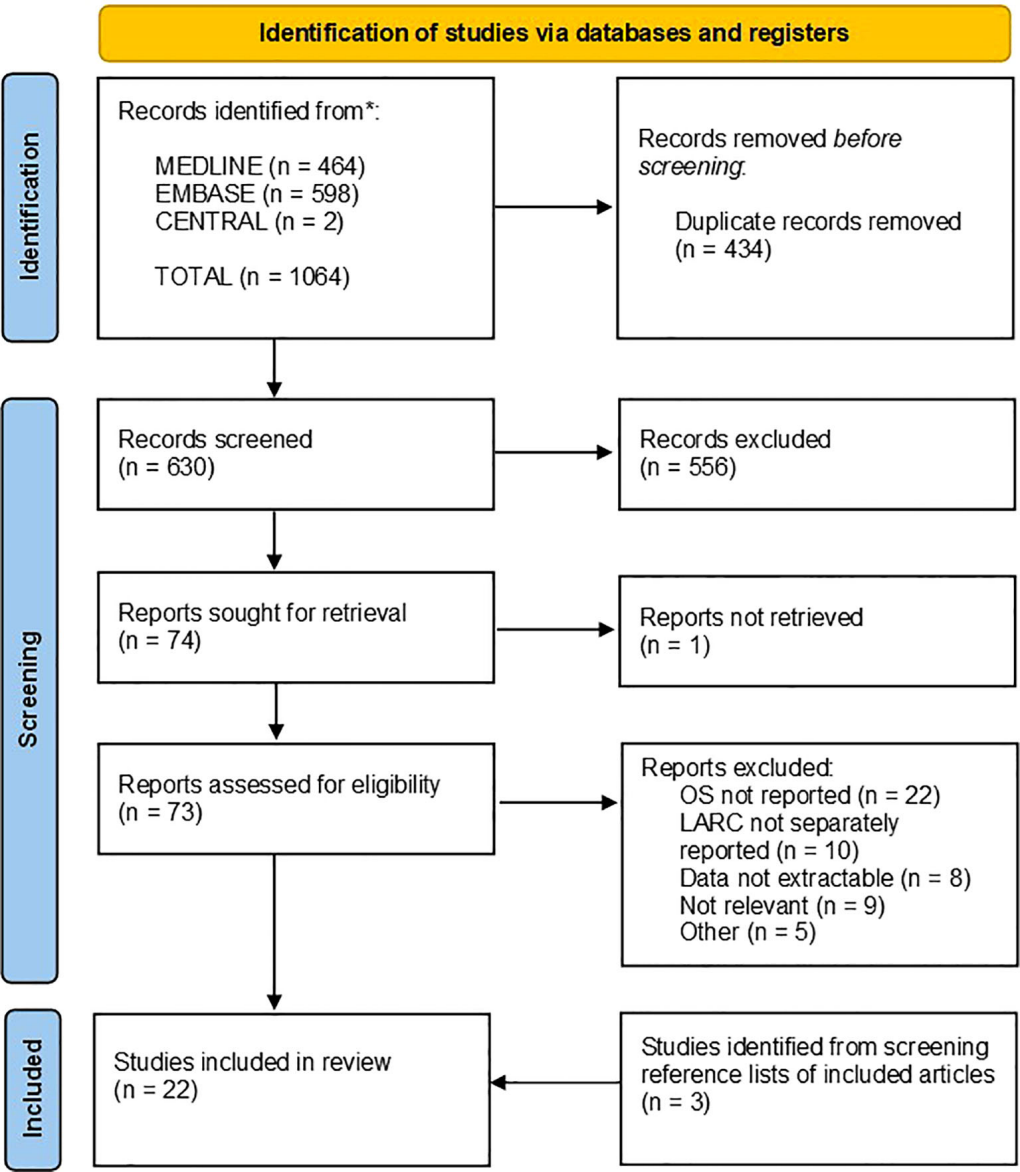
PRISMA flow chart showing search strategy and results. Searches were performed on the 8^th^ September 2023, with results limited to English language publications since 2003. Included articles reported the effect of at least one immune feature on overall survival in LARC patients. A table of articles which were excluded at the full-text screening stage is presented in **Supplementary Table 4** along with detailed reasons for exclusion. A total of twenty-two articles were included, three of which were identified by recursively screening reference lists of included articles.

The final, comprehensive database searches were performed on the 8^th^ September 2023. 464, 598 and 2 records were identified from MEDLINE, EMBASE and CENTRAL respectively. The PRISMA flowchart is presented in **Figure 1**. References were exported to Zotero 6.0.30, and duplicate records were flagged automatically. Suspected duplicate records were manually reviewed before 434 were excluded. 630 abstracts were screened, of which 556 were excluded. 73 full text articles were assessed against the eligibility criteria, of which 54 were excluded (17–70). 3 additional studies were identified from reference lists of included articles. Finally, 22 articles were included (presented in **Table 1**). A full list of excluded articles with reasons for exclusion is provided in **Supplementary Table 4**.

**Table 1:** Studies included in systematic review.

First author	Year	Journal	Country	Institution	Study design	Start date	End date	Definition of LARC	Inclusion criteria	Exclusion criteria	Patients (n)
Huang (90)	2021	Oncoimmunology	Taiwan	China Medical University Hospital	Retrospective observational cohort	Jan-06	Dec-14	Not further described	LARC, nCRT	Inadequate follow-up data	70
Shao (87)	2017	Cancer Manag Res	China	Fujian Medical University Cancer Hospital	Retrospective observational cohort	Jun-06	Feb-15	cT3–4 or N+	LARC, nRT or nCRT	Synchronous metastasis, concurrent malignancy, pCR, inadequate or unavailable specimens	68
Zhang (80)	2019	Cancer Manag Res	China	Fujian Medical University Cancer Hospital	Retrospective observational cohort	Feb-12	Sep-15	cT3–4 or N+	LARC, nRT or nCRT	synchronous malignancy, Karnofsky performance score <70, inadequate specimens	76
Boustani (85)	2020	Cells	France	Georges-Francois Leclerc Cancer Center	Retrospective observational cohort	Jan-95	Dec-07	Not further described	LARC, treatment with curative intent, nRT or nCRT, TME, available specimens	Inadequate specimens	74
Chen (76)	2019	J. Cancer Res. Clin. Oncol.	Taiwan	China Medical University Hospital	Retrospective observational cohort	Jan-06	Dec-14	cT3–4 or N+	LARC, nCRT		171
Chiang (86)	2019	Cancer Immunol. Immunother.	Taiwan	China Medical University Hospital	Retrospective observational cohort	Jan-06	Dec-13	cT3–4 or N+	LARC, nCRT		104
Schollbach (81)	2019	Cancer Immunol. Immunother.	Germany	University Hospital of Würzburg	Retrospective observational cohort	May-09	Mar-15	uT3–4 or N+, M0	LARC, mid- or low- rectum	synchronous metastasis, follow up < 1yr, pCR	106
Alderdice (83)	2017	Mod Pathol	Northern Ireland	Queens University Belfast, Grampian Biorepository	Retrospective observational cohort			Not further described	LARC, nCRT	pCR, inadequate specimens	150
Chiang (77)	2021	Cancer Immunol. Immunother.	Taiwan	China Medical University Hospital	Retrospective observational cohort	Jan-06	Dec-14	cT3–4 or N+	LARC, nCRT, radical resection		211
Liu (82)	2021	OncoTargets Ther	China	Fujian Medical University Union Hospital	Retrospective observational cohort	Jan-11	Dec-15	Not further described	LARC, nCRT, radical resection	synchronous malignancy, Karnofsky performance score <70, inadequate specimens	191
Mirjolet (78)	2018	Oncoimmunology	France	Multicentre	Retrospective observational cohort	Jan-95	Dec-07	Not further described	LARC, nRT or nCRT	Inadequate specimens	237
Su (72)	2021	Ther. Radiol. Oncol.	Taiwan	MacKay Memorial Hospital	Retrospective observational cohort	Sep-05	Mar-18	cT3–4 or N+	LARC, surgery, adjuvant CRT	Inadequate specimens	55
Huang (73)	2022	J. Clin. Med.	Taiwan	MacKay Memorial Hospital	Retrospective observational cohort	Sep-10	Aug-17	cT3–4 or N+	LARC, nCRT, ECOG performance status 0-2, TME	pCR	69
Sissy (71)	2020	Clin. Cancer Res.	France	Multicentre	Retrospective observational cohort	Jan-99	Dec-16	Not further described	LARC, neoadjuvant treatment, TME, biopsies available		249
Peng (74)	2021	Neoplasma	China	The First Hospital of Quanzhou Affiliated to Fujian Medical University	Retrospective observational cohort			cT3–4 or N+	LARC, nCRT, Karnofsky performance status >70	Synchronous malignancy, immune disorder, incomplete data, or inadequate specimen	76
Teng (79)	2015	Transl Res.	China	Shandong Cancer Hospital and Institute	Retrospective observational cohort	Jan-00	Dec-10	Not further described	LARC, nCRT, radical resection		62
Lim (89)	2017	Int. J. Radiat. Oncol. Biol. Phys	Korea	Seoul National University College of Medicine	Retrospective observational cohort	Jan-05	Dec-12	Not further described	LARC, nCRT, available specimens, complete clinicopathological data, cM0, TME		123
Sato (84)	2014	PLoS One	Japan	Gunma University Graduate School of Medicine	Retrospective observational cohort	Jan-03	Dec-11	N+	LARC, nHCRT, specimens available		56
Huang (92)	2018	Cancer Immunol. Immunother.	Taiwan	China Medical University Hospital	Retrospective observational cohort	Jan-06	Jan-13	cT3–4 or N+	LARC, nCRT, radical resection	M1, IBD, synchronous disease, hereditary cancer syndrome, previous cancer or pelvic radiotherapy, emergency presentation	89
Lim (91)	2022	Front. Oncol	Korea	Seoul National University College of Medicine	Retrospective observational cohort			cT3–4 or N+	LARC, nCRT, specimens available, M0		165
Jarosch (75)	2018	Oncoimmunology	Germany	University Hospital Carl Gustav Carus	Retrospective observational cohort	Jan-01	Dec-13	Not further described	LARC, nCRT		130
Saigusa (88)	2016	Int J Clin Oncol	Japan	Mie University Graduate School of Medicine	Retrospective observational cohort	Aug-03	May-14	Not further described	LARC, nCRT	pCR, inadequate specimens	90

Table showing study metadata for the 22 included articles. One study was a multicentre observational cohort; the remainder were single centre cohort studies. Included studies originated from 15 centres across 7 countries. Patients were included from January 1995 to March 2018.

### 3.2 Assessment of risk of bias

**Figure 2:**
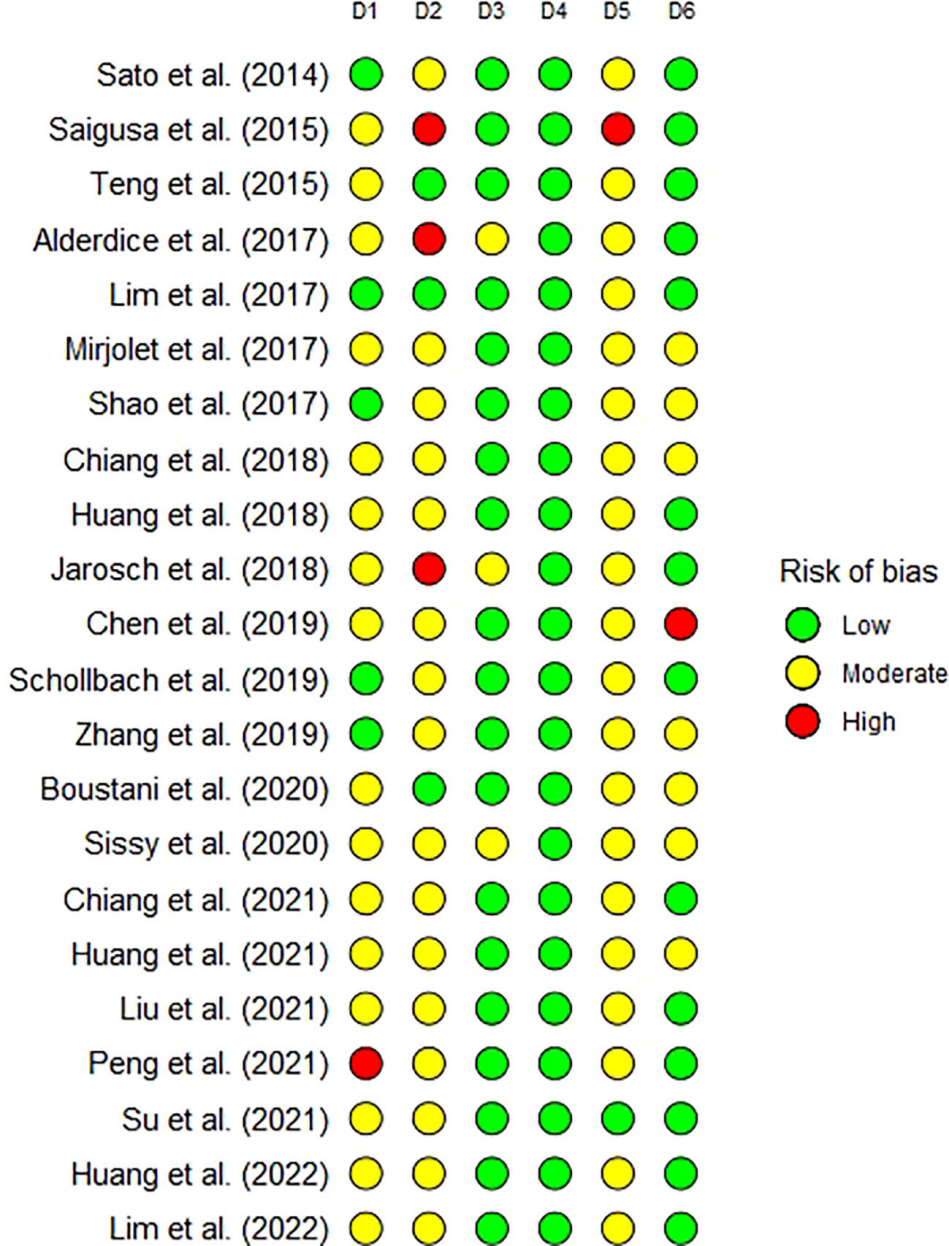
The risk of bias of individual studies as assessed by the Quality in Prognosis Studies (QUIPS) tool which includes six domains: D1 - participation; D2 - attrition; D3 - prognostic factor measurement; D4 - outcome measurement; D5 - confounding; and D6 - analysis and reporting.

The risk of bias assessments of included studies are presented in **Figure 2**. The risk of bias was concentrated in the participation, attrition, and confounding domains. The principal sources of risk of bias were a lack of clarity on whether consecutive patients were recruited, a low proportion of otherwise suitable patients having available specimens, early loss to follow up, and not reporting or controlling for resection margin status.

### 3.3 Definitions of LARC

22 studies included patients with LARC, of which ten did not provide a further description or definition of LARC. LARC was defined as cT3-4/N+ rectal cancer by 11 studies, and as N+ by one study. No studies included patients with LRRC. No studies reported a T4 specific subgroup.

### 3.4 Immune prognostic factors

Complete hazard ratios and confidence intervals extracted from each paper are presented in **Supplementary Table 5**. A hazard ratio of less than 1 reflects a lower chance of mortality in that group compared to the reference group.

#### 3.4.1 T-lymphocytes

One study reported the effect of a biopsy-adapted immunoscore (calculated as the mean of CD3 and CD8 staining intensity percentiles and categorised into high, intermediate and low) on overall survival in LARC (71). A high immunoscore in pre-neoadjuvant treatment biopsies was associated with greater survival compared to low (HR 0.38, 0.15-0.98). Comparisons with the intermediate group in the form of hazard ratios are not reported, but Kaplan-Meier survival analysis showed the survival in the intermediate group was between that of the high and low groups.

Two studies reported the association of lymphocyte response with overall survival in LARC (72, 73). Both studies assessed the intra-tumoral and peri-tumoral lymphocyte response (ILR and PLR) on haematoxylin and eosin stained surgical specimens of LARC patients, including those who did not (72) and did (73) undergo neoadjuvant chemoradiotherapy respectively. The absent lymphocyte response (ILR-/PLR-) showed a trend towards worse overall survival in patients not undergoing neoadjuvant treatment, although this was not significant. In patients undergoing neoadjuvant treatment there was no association of ILR with overall survival, and a trend towards improved survival in the PLR+ group although again, this was not significant. The heterogeneity in treatment given precludes meta-analysis.

Two studies reported on the influence of T-lymphocytic infiltration measured using CD3 immunohistochemistry (74, 75). The proportion of CD3+ cells (74) was not associated with overall survival in LARC patients undergoing neoadjuvant treatment. Neither epithelial nor stromal compartment CD3 staining (75) was associated with overall survival in LARC patients undergoing neoadjuvant chemoradiotherapy, on adjusted analysis (hazard ratios from unadjusted analysis were not available).

**Figure 3:**
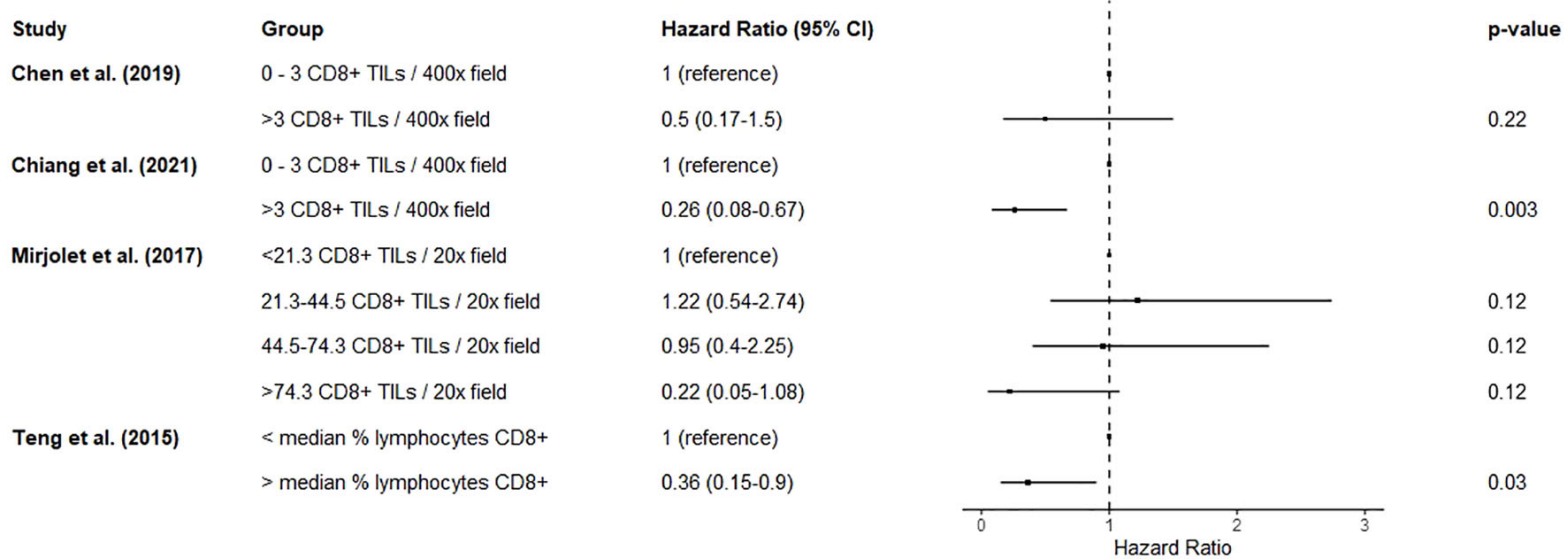
Forest plot summarizing the effect of biopsy CD8+ cells on mortality. Hazard ratios < 1 suggest improved overall survival. Chen et al. (2019) and Chiang et al. (2021) originated from the same centre and report overlapping cohorts. There was substantial heterogeneity in measurement and classification methods for this prognostic factor, so a pooled estimate was not calculated as it would not be meaningful.

Four studies described the association of CD8+ lymphocytic infiltration in pre-treatment biopsies with overall survival (76–79). Two of these (76, 77) reported a highly overlapping cohort for this prognostic factor, so the smaller cohort (76) was not included in the analysis for this prognostic factor. >3 CD8+ tumour infiltrating lymphocytes per high-power field was associated with improved overall survival (HR 0.26, 0.08-0.67) (77). Another of these studies demonstrated an apparent dose response relationship between increasing numbers of CD8+ tumour infiltrating lymphocytes per field and better survival (78), although this did not reach significance. Increased proportion of lymphocytes expressing CD8 was associated with greater overall survival (HR 0.36, 0.15-0.90) (79). Unfortunately, due to the differing measurements of CD8+ lymphocytes used, a pooled estimate would not be meaningful. A forest plot of results without a pooled estimate is shown in **Figure 3**.

**Figure 4:**
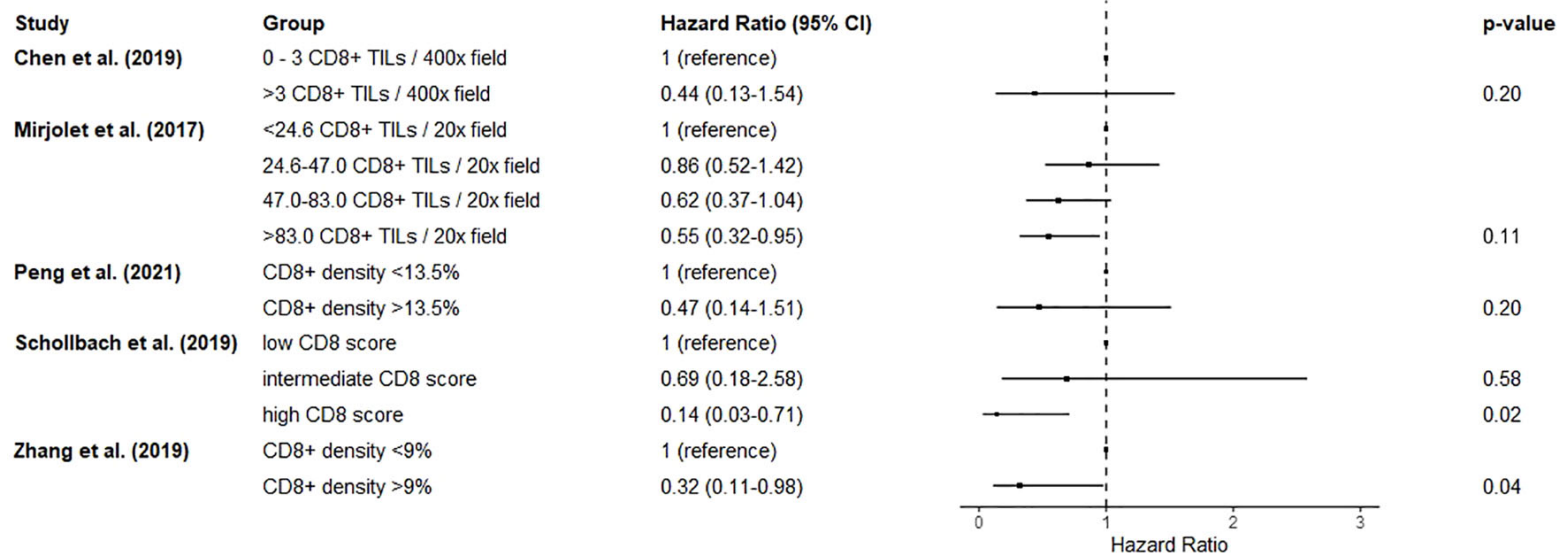
Forest plot showing the effect of resection specimen CD8+ cells on mortality. Hazard ratios < 1 suggest improved overall survival. There was substantial heterogeneity in measurement and classification methods for this prognostic factor, so a pooled estimate was not calculated as it would not be meaningful.

Six studies reported on the effect of CD8+ lymphocytic infiltration in post-neoadjuvant treatment specimens on overall survival (74–76, 78, 80, 81). Two studies reported on the proportion of lymphocytes expressing CD8, using cut-offs of 9% (80) and 13.5% (74) respectively. At the lower cut-off, a high proportion of lymphocytes expressing CD8 was associated with improved overall survival (HR 0.32, 0.11-0.98) (80), whereas in the higher cut-off cohort CD8 density was not significantly associated with survival (74). More than three CD8+ tumour infiltrating lymphocytes per high-power field was not significantly associated with survival (76). Two studies reported a dose response relationship between groups with increasing numbers of CD8+ cells and improved overall survival (78, 81), although significance was only reached where the highest CD8 groups were compared with the reference groups (HR 0.14, 0.03-0.71 (81) and HR 0.55, 0.32-0.95 (78) respectively). Comparisons of intermediate groups were not significant. In the final study neither stromal or epithelial CD8+ cells were significantly associated with survival (75). Due to heterogeneity in prognostic factor measurement, a forest plot of results without quantitative synthesis is shown in **Figure 4**.

A single study reported on the influence of CD4+ lymphocytic infiltration on overall survival (79). This study concluded that higher proportion of lymphocytes expressing CD4 in LARC biopsies was not associated with overall survival.

Two studies reported on the association of FoxP3 expression with overall survival (78, 79). The number of FoxP3 expressing lymphocytes in pre-treatment biopsies showed an unusual relationship with overall survival, where an intermediate number of FoxP3+ cells was shown to be protective (HR 0.44, 0.22-0.90) compared to the lower reference group, whereas there was no significant relationship for higher quartiles (78). A reduced number of FoxP3 expressing lymphocytes in the biopsy stromal compartment tended to be associated with inferior survival, although this was only significant in an intermediate group (HR 2.72, 1.29-5.73) (78). Both FoxP3+ cells in the biopsy epithelial compartment, and the ratio of CD8+ to FoxP3+ cells in the biopsy were not associated with survival. The surgical specimens showed no association of FoxP3 expressing lymphocytes, or the ratio of CD8+ to FoxP3+ cells with survival (78). Interestingly, the change in the ratio of CD8+ to FoxP3+ cells in the epithelial compartment between biopsy and resection was significantly associated with overall survival, with the quartile with the greatest reduction in the ratio showing the best survival (78). A similar relationship was not reported for the change in the number of FoxP3+ cells overall, or the ratio of CD8+ to FoxP3+ cells across epithelial and stromal compartments. In the other study, the numbers of FoxP3 expressing cells in pre-treatment biopsies was not associated with overall survival (79). As patients were grouped arbitrarily by FoxP3+ count by each study, meta-analysis would not be meaningful.

#### 3.4.2 Other cell types

A single study reported on the effect of macrophage associated markers with overall survival (82). Increased CD163+ and CD68+ cell count (representing M2 type macrophages and all tumour associated macrophages respectively) and increased MCSF and CCL2 expression, in both LARC biopsy and resection specimens, were associated with inferior overall survival.

Another study reviewed the association of myeloid derived suppressor cells (MDSC) with overall survival (79). The CD11b+/CD33+ MDSC count in pre-treatment biopsies was not significantly associated with overall survival (79).

One study reported on the influence of CD56 expression (a marker for natural killer cells) in post-neoadjuvant chemoradiotherapy specimens on overall survival in LARC (83). Positive CD56 expression (representing ≥4 CD56+ cells) was associated with better overall survival (HR 0.28, 0.11-0.73).

#### 3.4.3 Immune activity, checkpoints and exhaustion

One study reported on the effect of HLA class 1 expression in post-neoadjuvant hyperthermo-chemoradiotherapy specimens on overall survival (84). They concluded that HLA class 1 was not associated with overall survival.

**Figure 5:**
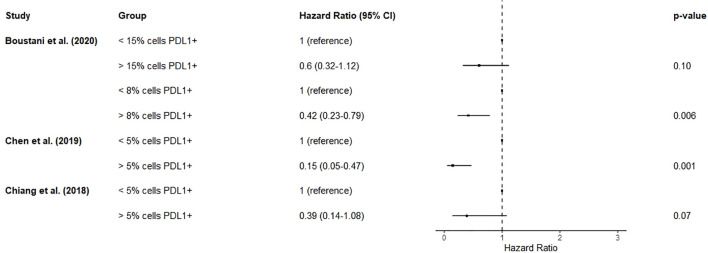
Forest plot detailing the effect of biopsy PD-L1 expression on mortality. Hazard ratios < 1 suggest improved overall survival. Chen et al. (2019) and Chiang et al. (2018) originated from the same centre and report overlapping cohorts. There were differing arbitrary cut-offs used to classify groups for this prognostic factor. A pooled estimate was not calculated as it would not be meaningful.

Three studies reported on the influence of PD-L1 expression in pre-neoadjuvant treatment biopsy specimens on overall survival (76, 85, 86). >8% of cells expressing PD-L1 was associated with improved overall survival (HR 0.42, 0.23-0.79) (85). The other two studies reported highly overlapping cohorts (76, 86), so the study with the smaller cohort (86) was excluded from the analysis for this prognostic factor. >5% of cells expressing PD-L1 was associated with greater survival (HR 0.15, 0.05-0.47) (76). A forest plot of results is shown in **Figure 5**.

**Figure 6:**
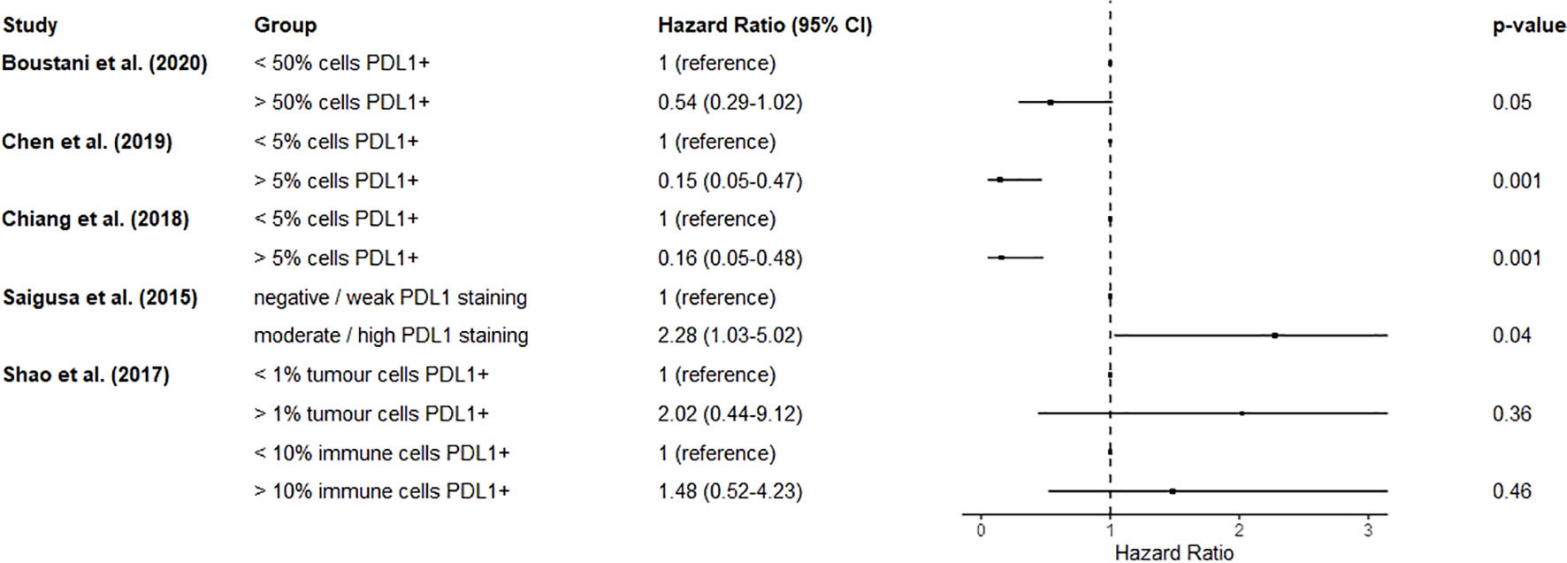
Forest plot summarizing the effect of resection specimen PD-L1 expression on mortality. Hazard ratios < 1 suggest improved overall survival. Chen et al. (2019) and Chiang et al. (2018) originated from the same centre and report overlapping cohorts. There were differing arbitrary cut-offs used to classify groups for this prognostic factor. A pooled estimate was not calculated as it would not be meaningful.

Five studies reported on the effect of PD-L1 expression in surgical specimens on overall survival (76, 85–88). Neither tumour cell nor immune cell PD-L1 expression was significantly associated with overall survival (87). >50% of cells expressing PD-L1 was also reported to be not associated with survival (85). As above, two studies reported highly overlapping cohorts (76, 86), so the study with the smaller cohort (86) was excluded from the analysis for this prognostic factor. It was reported that >5% of cells expressing PD-L1 in the surgical specimen was associated with greater survival (HR 0.15, 0.05-0.47) (76). However another study reported that greater PD-L1 staining intensity was associated with poorer overall survival (HR 2.28, 1.03-5.04) (88). A forest plot of results without a pooled estimate is shown in **Figure 6** due to differences in prognostic factor measurement.

Two studies reported on the influence of PD-L1 trajectory between biopsy and resection specimens on overall survival (85, 89). The absolute change in PD-L1 expression between biopsy and resection specimens was not significantly associated with survival (85). However, patients who had high PD-L1 expression in both biopsy and resection specimens demonstrated improved overall survival compared to all other groups (HR 0.32, 0.13-0.76) (85), and to those who had low PD-L1 expression in both biopsy and resection specimens (85, 89).

One study reported on the association between PD-L1 promoter methylation in post-neoadjuvant chemoradiotherapy specimens and overall survival in LARC (90). High methylation at two loci (cg15837913 and cg19724470) was associated with reduced overall survival (HR 2.68, 1.18-6.08 and HR 3.24, 1.03-10.2 respectively).

One study reported that PD-1 expression in resection specimens was not associated with survival (74).

One study reported the effect of a composite model enumerating risk factors including high numbers of MDSCs, the ratio of PD-1+ to CD8+ cells, and either PD-L1 expression on immune cells or tumour cells on overall survival (91). Both models showed that increasing numbers of risk factors was associated with reduced survival (HR 17.80, 3.76-84.24 and HR 7.07, 1.79-27.87 for each model respectively).

One study reported the association of a composite model including cytoplasmic expression of high mobility group box protein 1 (cyto-HMGB1) and PD-1+ tumour infiltrating lymphocytes on overall survival (92). No survival benefit was reported when either showed positive expression when compared to both being negatively expressed (92).

One study reported on the effect of CTLA-4 expression in biopsy specimens (79) and was unable to demonstrate association between CTLA-4 expression and survival.

One study reports on the influence of Granzyme B, a marker of cytotoxic lymphocyte activity, on overall survival (75). The absolute numbers of lymphocytes expressing both Granzyme B and CD8 in either the epithelial or stromal compartment were not associated with survival. However, an increased proportion of CD8+ lymphocytes expressing Granzyme B within the tumour epithelium was associated with improved survival on adjusted analysis (HR 0.51, 0.27-0.96) (75).

One study reported on the association of indoleamine 2,3-deoxygenase 1 (IDO1) expression on overall survival (81). IDO1 expression was not associated with survival (81).

One study explored the influence of a composite model including interferon gamma (IFN-γ) and tumour PD-L1 expression in both biopsy and resection specimens (86). High expression of both PD-L1 and IFN-γ in resection specimens (HR 0.51, 0.04-0.58) but not biopsy specimens was associated with improved survival (86).

One study reported on the effect of lymphocyte activation gene 3 (LAG-3) and T-cell immunoglobulin and mucin-domain containing 3 (TIM-3) on overall survival in specimens from patients who had undergone neoadjuvant treatment (74). Expression levels of either marker, on both tumour and immune cells was not associated with overall survival (74).

#### 3.4.4 Other features

One study investigated the effect of germline pattern recognition receptor polymorphisms on overall survival (77). The CC genotype (homozygous loss-of-function) of the formyl peptide receptor 1 (FPR1) was associated with significantly reduced overall survival (HR 2.41, 1.21-5.21) in LARC. Polymorphisms of TIM-3, P2X purinergic receptor 7 (P2RX7), and Toll-like receptor 1 (TLR1) were not associated with adverse outcomes.

## 4 Discussion

### 4.1 Immune prognostic factors in LARC

LARC and LRRC do not currently have validated novel biomarkers to inform prognosis, counselling of patients, case selection or follow up. We aimed to systematically review the literature on tumour immune microenvironmental prognostic features in LARC and LRRC. Here we identified 22 studies of immune prognostic features in LARC, and a complete absence of evidence of immune factors in LRRC. We reported findings that CD8+ lymphocyte and natural killer cell infiltration are associated with better outcomes in LARC. In contrast, we identified that infiltration of other lymphocyte subgroups is not prognostic. We found inconsistent evidence of an effect of PD-L1 expression on overall survival. We also identified M2 macrophage infiltration and germline FPR1 loss of function as negative prognostic features.

These findings suggest a role for both innate and acquired immunity in affecting outcomes following rectal cancer surgery. The findings suggest that immune escape of LARC is not predominantly mediated by increased infiltration of immunosuppressive or regulatory lymphocytes. Instead, there is a possible role for M2 macrophages in suppressing immune activity in the tumour or promoting metastasis. It is unclear how PD-L1 expression influences overall survival. Mechanistically, it might be expected that high tumour PD-L1 expression would allow tumour cells to evade killing by cytotoxic cells. However, high expression may also reflect greater immune activity and IFN-γ signalling in the tumour microenvironment (86). Methylation of the PD-L1 promoter was negatively correlated with PD-L1 expression and associated with worse survival. However, whether this reflects a hypermethylation phenotype or whether the prognostic effect was specifically related to suppression of PD-L1 would require further study. FPR1 is a widely expressed pattern recognition receptor involved in the detection of damage- and pathogen- associated molecular patterns as part of the innate immune system. The finding that homozygous germline loss of function of this receptor is associated with worse outcomes suggests a role for the innate pattern recognition in initiating an immune response to LARC.

### 4.2 Commonly reported definitions of LARC

The most common definition of LARC used in the included studies, where it was reported, was AJCC stage II/III, i.e. either T3-4, or node positive (N+) disease. Pelvic MRI, the gold standard of imaging based rectal cancer staging, is relatively poor at assessing lymph node involvement (sensitivity of 77%) (93), and lymph nodes from the resection specimen may be affected by response to neoadjuvant treatment. Approximately 15% of T1 rectal cancers display lymph node metastasis (94, 95), which has been associated with an absence of lymphocytic infiltration (96). In patients undergoing neoadjuvant chemoradiotherapy, increasing risk of nodal involvement is associated with increasing γpT-stage, ranging from 7% in γpT0 to 46% in γpT4 disease (97). Invasion of adjacent organs is neither necessary nor sufficient for lymph node metastasis and likely represents a separate biological process. The presence of cancer within regional lymph nodes may also negatively affect their functioning as secondary lymphoid organs (98) and contribute to the immune evasion of the primary tumour.

Given the challenges with assessing lymph node involvement on MRI, and the plausibly separate biological processes underlying local invasion and nodal metastasis, it is questionable whether the commonly used definition of LARC – including patients with T1 N1 and T4 N0 disease in the same category – is appropriate. It remains unclear whether tumour immune microenvironment prognostic factors identified with a broad definition of LARC would generalize to patients requiring exenterative surgery.

### 4.3 Risk of bias

The common sources of potential bias in included studies were incomplete recruitment of patients due to missing data or inadequate specimens, early censoring due to loss to follow-up, and not explicitly reporting and controlling for resection margin status – a known prognostic marker. The risk of bias was typically moderate, and it is unclear from biological principles in which direction any bias would affect the results.

CD8+ cell infiltration was widely studied, had a consistent direction of effect, and showed a dose-response relationship to overall survival. It is unlikely that the association of CD8+ cells with improved overall survival would be vulnerable to bias. In contrast, the association of PD-L1 expression with improved survival was inconsistent and may be affected by bias. The associations of natural killer cells, M2 macrophages, and germline FPR1 receptor loss-of-function with survival outcomes were less well studied and deserve further scrutiny.

A degree of recruitment and attrition bias may be expected by the retrospective design of included studies. This could be mitigated by prospective studies requiring dedicated samples to be taken, using a standardized data collection tool and adopting protocolized follow-up. Similarly, reporting of resection margin status and other pathological features of interest may be improved using standardized pathology reporting templates in future studies.

### 4.4 Systematic review limitations

The studies reviewed included patients who underwent surgery between January 1995 and March 2018. Changes to surgical and oncological practice since then (including greater standardization and centralization of treatment, greater use of neoadjuvant and combination therapies, and personalized treatments) may limit generalization to current patients.

Case ascertainment in many of the studies may not have been complete as many patients were excluded as insufficient or inadequate specimens were available. This may introduce confounding if, for example, serial sectioning of specimens leading to their exhaustion was performed more frequently in patients with a higher risk of mortality, or a particular immune phenotype.

There was substantial heterogeneity in the methods used to measure immune microenvironmental features and subsequently classify patients into groups in the included studies. Studies variously reported densities or ratios of different cell types, with differing (often arbitrary or data-derived) cut-offs used to define groups. This heterogeneity in prognostic factor measurement prevented pooled estimates from being meaningfully calculated. Further work would benefit from standard methodology to measure immune cell infiltration or immune marker expression, to allow for comparison between studies and across time points.

Included studies originated from few geographic areas, concentrated in Europe and East Asia. Indeed, 5 of the included articles originated from the same centre: the China Medical University Hospital, Taichung. It is therefore unclear to what extent the results can be generalized internationally. Further research could seek to include collaboration between participating centres across continents.

### 4.5 Immunotherapy of LARC

The association of cytotoxic cell infiltration and PD-L1 expression with improved survival is consistent with an increasingly important role for immunotherapy in the treatment of LARC.

In resectable colon cancer, the NICHE study (99) enrolled 62 patients for treatment with neoadjuvant CTLA-4 and PD-1 blockade, and reported a 100% pathologic response rate (defined as > 50% tumour regression) in patients with mismatch-repair deficient (dMMR) disease. In patients with mismatch repair proficient (pMMR) resectable colon cancer, NICHE also demonstrated that the response to immunotherapy was predicted by CD8+ lymphocytic infiltration (100). Subsequent studies have demonstrated impressive response rates to neoadjuvant PD-1 and CTLA-4 blockade (NICHE-2) (101), and PD-1 and LAG-3 blockade (NICHE-3) (102) in 113 and 59 patients with dMMR locally-advanced colonic cancer respectively. As fewer than 10% of LARC are dMMR (103) the degree to which these findings might transform the treatment of LARC is currently limited.

In metastatic colorectal cancer, KEYNOTE-177 enrolled 307 patients with MSI-H/dMMR disease, and demonstrated no difference in overall survival, but fewer treatment related adverse effects in patients treated with PD-1 blockade with pembrolizumab versus chemotherapy (104). The reduction in adverse events may be used to explain the observed improvement in health-related quality of life in the intervention group (105). IMblaze370 investigated the use of PD-L1 inhibition with atezolizumab (with or without MEK inhibitor cobimetinib) versus monotherapy with the tyrosine kinase inhibitor regorafenib in 363 patients with previously treated irresectable locally advanced or metastatic colorectal cancer, finding no difference in overall survival between groups. (106). These studies might suggest that benefit from immunotherapy is limited to patients with resectable disease.

In the setting of rectal cancer specifically, cohort studies suggested a role for PD-1 blockade as part of neoadjuvant treatment, with patients with complete response potentially being suitable for a watchful waiting strategy (45, 107). The phase-II TORCH trial showed that PD-1 blockade is associated with high rates of pathological complete response to neoadjuvant chemoradiotherapy (108). The phase-II PRIME-RT trial (109) has recently reported a complete response rate of 52% in 42 patients undergoing PD-1 blockade with durvalumab in addition to total neoadjuvant treatment (110). It is currently unclear whether the immune prognostic features reported here also might predict responses to immunotherapy.

## 5 Conclusion

Infiltration of cytotoxic cells is associated with improved survival in LARC. There is inconsistent evidence of a relationship between PD-L1 expression and improved outcomes. M2 macrophage infiltration and germline loss of function of the FPR1 receptor have been associated with worse survival but deserve further investigation. Future work should prospectively validate these findings using standardized methodology and attempt to uncover the mechanisms underlying improved survival in LARC.

## Data Availability

The original contributions presented in the study are included in the article/**Supplementary Material**. Further inquiries can be directed to the corresponding author.
