# Supplementary material for: Tumour immune microenvironment prognostic factors in locally advanced rectal cancer, a systematic review
Source: Front Oncol. 2025 Dec 19;15:1688696. doi: 10.3389/fonc.2025.1688696 (PMC12757241; doi:10.3389/fonc.2025.1688696)
Supplement: Supplementary file 1 [file DataSheet1.docx]

**Supplementary table 1.**

**MEDLINE search strategy**

| # | searches |
| --- | --- |
| 1 | rectal neoplasms/ |
| 2 | ((rect* or rectal or rectum) adj3 (adenocarcinoma or cancer* or carcinoma* or malignan* or neoplas* or oncolog* or tumo?r*)).ti,ab,kw,kf. |
| 3 | 1 or 2 |
| 4 | (local* adj3 advanc*).ti,ab,kw,kf. |
| 5 | (local* adj3 recur*).ti,ab,kw,kf. |
| 6 | 4 or 5 |
| 7 | 3 and 6 |
| 8 | immune system phenomena/ |
| 9 | tumor microenvironment/ |
| 10 | (immun* or inflamm* or lympho* or macroph* or granulo* or ?????ophil or T?cell or B?cell or dendritic or TIL or TAM or PD-??1 or CTLA?4 or MHC).ti,ab,kw,kf. |
| 11 | 8 or 9 or 10 |
| 12 | 7 and 11 |
| 13 | (mort* or surv*).ti,ab,kw,kf. |
| 14 | recurren*.ti,ab,kw,kf. |
| 15 | (metasta* or ((dist* or local*) adj3 recurren*)).ti,ab,kw,kf. |
| 16 | 13 or 14 or 15 |
| 17 | 12 and 16 |
| 18 | letter/ |
| 19 | editorial/ |
| 20 | news/ |
| 21 | exp historical article/ |
| 22 | anecdotes as topic/ |
| 23 | (letter or comment*).ti |
| 24 | (abstract or comment or conference or letter).pt. |
| 25 | 18 or 19 or 20 or 21 or 22 or 23 or 24 |
| 26 | 17 not 25 |
| 27 | limit 26 to english language |
| 28 | limit 27 to yr="2003-Current" |
| 29 | limit 28 to humans |

**Supplementary table 2.**

**EMBASE search strategy**

| # | searches |
| --- | --- |
| 1 | rectum tumor/ |
| 2 | ((rect* or rectum or rectal) adj3 (adenocarcinoma or cancer* or carcinoma* or malignan* or neoplas* or oncolog* or tumo*r)).ti,ab,kw,kf. |
| 3 | 1 or 2 |
| 4 | (local* adj3 advanc*).ti,ab,kw,kf. |
| 5 | (local* adj3 recur*).ti,ab,kw,kf. |
| 6 | 4 or 5 |
| 7 | 3 and 6 |
| 8 | immune system/ |
| 9 | tumor microenvironment/ |
| 10 | (immun* or inflamm* or lympho* or macroph* or granulo* or ?????ophil or T?cell or B?cell or dendritic or TIL or TAM or PD-??1 or CTLA?4 or MHC).ti,ab,kw,kf. |
| 11 | 8 or 9 or 10 |
| 12 | 7 and 11 |
| 13 | (mort* or surv*).ti,ab,kw,kf. |
| 14 | recurren*.ti,ab,kw,kf. |
| 15 | (metasta* or ((dist* or local*) adj3 recurren*)).ti,ab,kw,kf. |
| 16 | 13 or 14 or 15 |
| 17 | 12 and 16 |
| 18 | letter/ |
| 19 | editorial/ |
| 20 | news/ |
| 21 | exp "history of medicine"/ or exp history/ |
| 22 | literature/ |
| 23 | (letter or comment*).ti |
| 24 | (abstract or comment or conference or letter).pt. |
| 25 | 18 or 19 or 20 or 21 or 22 or 23 or 24 |
| 26 | 17 not 25 |
| 27 | limit 26 to english language |
| 28 | limit 27 to yr="2003-Current" |
| 29 | limit 28 to human |

**Supplementary table 3.**

**Central search strategy**

| # | Search |
| --- | --- |
| 1 | rectal neoplasm |
| 2 | locally advanced:ti,ab,kw |
| 3 | locally recurrent:ti,ab,kw |
| 4 | #1 AND (#2 OR #3) |
| 5 | (immune system) OR (tumor microenvironment) |
| 6 | #4 and #5 |
| 7 | (mortality) OR (survival) OR (metastasis) OR (recurrence) |
| 8 | #6 AND #7 |

**Supplementary table 4.**

**List of excluded full text articles**

| **First author** | **Year** | **Journal** | **DOI** | **Country** | **Institution** | **Reason for exclusion** |
| --- | --- | --- | --- | --- | --- | --- |
| Feng et al. | 2022 | Discover Oncology | 10.1007/s12672-022-00471-8 | China | Zhejiang Cancer Hospital | Overall survival not extractable |
| Wang et al. | 2015 | International Journal of Colorectal Disease | 10.1007/s00384-015-2226-6 | China | Peking University Cancer Hospital and Institute | Overall survival not reported |
| Ji et al. | 2018 | Cancer Immunology Research | 10.1158/2326-6066.CIR-17-0630 | China | Peking University Cancer Hospital and Institute | Overall survival not extractable |
| Kim et al. | 2021 | Oncology Letters | 10.3892/ol.2021.12969 | Korea | Chungnam National University | Overall survival not extractable |
| Sütcüoğlu et al. | 2023 | Journal of Oncological Science | 10.37047/jos.2022-94374 | Turkey | Gazi University Faculty of Medicine | Overall survival not reported |
| Sawada et al. | 2021 | Annals of Surgical Oncology | 10.1245/s10434-021-09975-z | Japan | Cancer Institute Hospital | Overall survival not extractable |
| Takasu et al. | 2022 | BMC Cancer | 10.1186/s12885-022-10357-1 | Japan | University of Tokushima | LARC not separately reported |
| Ji et al. | 2020 | Journal for the Immunotherapy of Cancer | 10.1136/jitc-2020-000826 | China | Peking University Cancer Hospital & Institute | Overall survival not extractable |
| Xue et al. | 2022 | Frontiers in Oncology | 10.3389/fonc.2022.993726 | China | First Affiliated Hospital of Gannan Medical University | Overall survival not reported |
| Farchoukh et al. | 2021 | Modern Pathology | 10.1038/s41379-020-0619-8 | USA | University of Pittsburgh Medical Center | Overall survival not reported |
| Takahashi et al. | 2022 | Journal of Pathology: Clinical Research | 10.1002/cjp2.285 | Japan | Kitasato University School of Medicine | Overall survival not extractable |
| Akiyoshi et al. | 2019 | British Journal of Surgery | 10.1002/bjs.11179 | Japan | Cancer Institute Hospital | Overall survival not reported |
| Hasnis et al. | 2020 | Frontiers in Oncology | 10.3389/fonc.2020.01375 | Israel | Rambam HealthCare Campus | Overall survival not extractable |
| Tse et al. | 2023 | Oncoimmunology | 10.1080/2162402X.2023.2238506 | Australia | The University of Sydney | Overall survival not reported |
| Tominaga et al. | 2019 | PLoS ONE | 10.1371/journal.pone.0212978 | Japan | Cancer Institute Hospital | Overall survival not reported |
| Schollbach et al. | 2020 | International Journal of Colorectal Disease | 10.1007/s00384-019-03466-0 | Germany | University Hospital of Würzburg | Subgroup analysis of paper already included |
| Micev et al. | 2004 | Acta Chirurgica Iugoslavica | 10.2298/ACI0402099M | Serbia | Clinical Centre of Serbia | No methodology provided in article |
| D’Alterio et al. | 2014 | International Journal of Cancer | 10.1002/ijc.28689 | Italy | Istituto Nazionale per lo Studio e la Cura dei Tumori | Overall survival not reported |
| Chang et al. | 2012 | Modern Pathology | 10.1038/modpathol.2012.61 | USA | Stanford University | LARC not separately reported |
| De Bruin et al. | 2007 | Apoptosis | 10.1007/s10495-007-0088-2 | The Netherlands | Leiden University Medical Center | LARC not separately reported |
| De Bruin et al. | 2008 | Clinical Cancer Research | 10.1158/1078-0432.CCR-07-1597 | The Netherlands | Leiden University Medical Center | LARC not separately reported |
| Szynglarewicz et al. | 2008 | Journal of Gastrointestinal and Liver Diseases | Unavailable | Poland | Lower Silesian Oncology Center | LARC not separately reported |
| Lim et al. | 2014 | Anticancer Research | Unavailable | Australia | Liverpool Hospital | Overall survival not reported |
| Kirilovsky et al. | 2022 | Oncotarget | 10.18632/oncotarget.28100 | France | Laboratory of Integrative Cancer Immunology | Review article |
| Shabo et al. | 2009 | International Journal of Cancer | 10.1002/ijc.24506 | Sweden | Institution of Clinical and Experimental Medicine | LARC not separately reported |
| Zhou et al. | 2022 | Frontiers in Immunology | 10.3389/fimmu.2022.1050721 | China | Fujian Cancer Hospital | Prognostic factor not immune related |
| Akiyoshi et al. | 2021 | Cancer Immunology, Immunotherapy | 10.1007/s00262-020-02705-6 | Japan | Cancer Institute Hospital | Overall survival not reported |
| Oi et al. | 2021 | In Vivo | 10.21873/INVIVO.12276 | Japan | Dokkyo Medical University | Prognostic factor not immune related |
| Szynglarewicz et al. | 2006 | Gastroenterologia Polska | Unavailable | Poland | Lower Silesian Oncology Center | Full text not retrievable |
| Spolverato et al. | 2023 | International Journal of Surgery | 10.1097/JS9.0000000000000214 | Italy | Multicentre | LARC not separately reported |
| Matsutani et al. | 2018 | Cancer Science | 10.1111/cas.13542 | Japan | Osaka City University Graduate School of Medicine | Overall survival not extractable |
| Wu et al. | 2018 | Medicine | 10.1097/MD.0000000000011972 | China | Mudanjiang City Xinxiang Medical University | Overall survival not reported |
| Cho et al. | 2022 | Oncoimmunology | 10.1080/2162402X.2022.2148374 | Korea | University of Ulsan College of Medicine | Overall survival not reported |
| Zlobec et al. | 2008 | British Journal of Cancer | 10.1038/sj.bjc.6604729 | Switzerland | University Hospital of Basel | LARC not reported |
| Vlems et al. | 2004 | Virchows Archiv | 10.1007/s00428-004-1033-1 | The Netherlands | UMC Nijmegen | LARC not separately reported |
| Sclafani et al. | 2014 | Clinical Cancer Research | 10.1158/1078-0432.CCR-14-0674 | International | Multicentre | LARC not reported |
| Richter et al. | 2017 | Journal of B.U.ON. | Unavailable | Czech Republic | Regional Hospital Liberec | Prognostic factor unable to be evaluated |
| Chen et al. | 2020 | Clinical Colorectal Cancer | 10.1016/j.clcc.2020.04.002 | Taiwan | Koo Foundation Sun Yat-Sen Cancer Center | Overall survival not reported |
| Sprenger et al. | 2013 | Cancer | 10.1002/cncr.27703 | Germany | University Medical Center | Prognostic factor not immune related |
| Deng et al. | 2014 | British Journal of Cancer | 10.1038/bjc.2013.767 | China | Sun Yat-sen University | Prognostic factor not immune related |
| Xu et al. | 2023 | Cancer Letters | 10.1016/j.canlet.2023.216145 | China | Zhejiang University School of Medicine | No data comparing immune prognostic factor to overall survival |
| Serna et al. | 2020 | Annals of Oncology | 10.1016/j.annonc.2020.06.003 | Spain | Vall d'Hebron Institute of Oncology | No data comparing immune prognostic factor to overall survival |
| Tayshetye et al. | 2023 | Cancers | 10.3390/cancers15010276 | USA | Allegheny Health Network | Overall survival not reported |
| Sadahiro et al. | 2010 | Anticancer Research | Unavailable | Japan | Tokai University School of Medicine | Overall survival not reported |
| Randrian et al | 2021 | Frontiers in Immunology | 10.3389/fimmu.2021.750407 | France | CHU Poitiers | LARC not separately reported |
| Chatila et al. | 2022 | Nature Medicine | 10.1038/s41591-022-01930-z | USA | Memorial Sloan Kettering Cancer Center | Overall survival not reported |
| Kamran et al. | 2019 | Clinical Cancer Research | 10.1158/1078-0432.CCR-19-0908 | USA | Massachusetts General Hospital | No data comparing immune prognostic factor to overall survival |
| Saigusa et al. | 2012 | International Journal of Oncology | 10.3892/ijo.2012.1598 | Japan | Mie University Graduate School of Medicine | Prognostic factor not immune related |
| Shinto et al. | 2020 | BJS Open | 10.1002/bjs5.50251 | Japan | National Defense Medical College | No data comparing immune prognostic factor to overall survival |
| Iseas et al. | 2021 | Frontiers in Oncology | 10.3389/fonc.2021.801880 | Argentina | Gastroenterology Hospital "Dr. Carlos Bonorino Udaondo" | Immune prognostic factor part of large composite measure with no other immune components |
| Gunther et al. | 2003 | International Journal of Colorectal Disease | 10.1007/s00384-002-0470-z | Germany | University of Erlangen | LARC not separately reported |
| Liu et al. | 2019 | Radiotherapy and Oncology | 10.1016/j.radonc.2018.12.001 | China | Southern Medical University, Guangzhou | No data comparing immune prognostic factor to overall survival |
| Jao et al. | 2012 | Annals of Surgical Oncology | 10.1245/s10434-012-2394-3 | Taiwan | National Defense Medical Centre and Tri-Service General Hospital | Prognostic factor not immune related |
| Wang et al. | 2022 | European Journal of Cancer | 10.1016/j.ejca.2022.07.016 | China | Sun Yat-sen University Cancer Center | No immune prognostic factor (single-arm trial of PD-1 blockade) |

**Supplementary table 5.**

**Hazard ratios and confidence intervals extracted from included papers**

| **Section** | **Author** | **Year** | **Specimen** | **Immune factor** | **Hazard ratio (95% c.i.)** | **p-value** |  |
| --- | --- | --- | --- | --- | --- | --- | --- |
| T-lymphocytes | El Sissy | 2020 | Biopsy | Biopsy-adapted immunoscore (high vs low) | 0.38 (0.15 - 0.98) | < 0.05 | * |
|  | Su | 2021 | Resection | ILR/PLR (any positive vs both negative) | 0.28 (0.08 - 1.07) | 0.06 |  |
|  | Huang | 2022 | Resection | ILR (positive vs negative) | 0.98 (0.38 - 2.53) | 0.97 |  |
|  |  |  | Resection | PLR (positive vs negative) | 0.46 (0.19 - 1.09) | 0.08 |  |
|  | Peng | 2021 | Resection | Proportion of cells CD3+ (> 12.5% vs < 12.5%) | 0.51 (0.16 - 1.61) | 0.25 |  |
|  |  |  | Resection | Proportion of cells CD8+ (> 13.5% vs < 13.5%) | 0.47 (0.15 - 1.51) | 0.20 |  |
|  | Jarosch | 2018 | Resection | Proportion of CD8+ cells that are GrzB+ | 0.51 (0.27 - 0.96) | < 0.05 | * |
|  | Chen | 2019 | Biopsy | CD8+ cells / 400x field (> 3 vs 0-3) | 0.50 (0.17 - 1.50) | 0.22 |  |
|  |  |  | Resection | CD8+ cells / 400x field (> 3 vs 0-3) | 0.44 (0.13 - 1.54) | 0.20 |  |
|  | Chiang | 2021 | Biopsy | CD8+ cells / 400x field (> 3 vs 0-3) | 0.26 (0.08 - 0.67) | < 0.01 | ** |
|  | Mirjolet | 2018 | Biopsy | CD8+ count (third vs. fourth quartile) | 1.22 (0.54 - 2.74) | 0.12 |  |
|  |  |  | Biopsy | CD8+ count (second vs. fourth quartile) | 0.95 (0.40 - 2.25) | 0.12 |  |
|  |  |  | Biopsy | CD8+ count (first vs fourth quartile) | 0.22 (0.05 - 1.08) | 0.12 |  |
|  |  |  | Resection | CD8+ count (third vs. fourth quartile) | 0.86 (0.52 - 1.42) | 0.11 |  |
|  |  |  | Resection | CD8+ count (second vs. fourth quartile) | 0.62 (0.37 - 1.04) | 0.11 |  |
|  |  |  | Resection | CD8+ count (first vs fourth quartile) | 0.55 (0.32 - 0.95) | 0.11 |  |
|  |  |  | Biopsy | FOXP3+ count (third vs fourth quartile) | 0.44 (0.22 - 0.90) | < 0.05 | * |
|  |  |  | Biopsy | FOXP3+ count (second vs fourth quartile) | 1.10 (0.61 - 2.00) | 0.02 |  |
|  |  |  | Biopsy | FOXP3+ count (first vs fourth quartile) | 0.48 (0.23 - 1.00) | 0.02 |  |
|  |  |  | Resection | FOXP3+ count (third vs fourth quartile) | 0.72 (0.43 - 1.20) | 0.05 |  |
|  |  |  | Resection | FOXP3+ count (second vs fourth quartile) | 0.67 (0.39 - 1.12) | 0.05 |  |
|  |  |  | Resection | FOXP3+ count (first vs fourth quartile) | 0.46 (0.27 - 0.80) | 0.05 |  |
|  | Teng | 2015 | Resection | Proportion of lymphocytes CD8+ (high vs low) | 0.36 (0.15 - 0.90) | < 0.05 | * |
|  |  |  | Resection | Proportion of lymphocytes CD4+ (high vs low) | 0.55 (0.24 - 1.30) | 0.17 |  |
|  |  |  | Resection | FOXP3+ cell count (high vs low) | 1.07 (0.47 - 2.49) | 0.86 |  |
|  | Zhang | 2019 | Resection | Proportion of lymphocytes CD8+ (>9% vs <9%) | 0.32 (0.11 - 0.98) | < 0.05 | * |
|  | Schollbach | 2019 | Resection | CD8 score (intermediate vs low) | 0.69 (0.19 - 2.58) | 0.58 |  |
|  |  |  | Resection | CD8 score (high vs low) | 0.14 (0.03 - 0.71) | < 0.05 | * |
| Other cell types | Liu | 2021 | Biopsy | CD163+ count (high vs low) | 1.01 (1.01 - 1.01) | < 0.01 | ** |
|  |  |  | Biopsy | MCSF intensity (high vs low) | 3.37 (1.99 - 5.72) | < 0.01 | ** |
|  |  |  | Biopsy | CCL2 intensity (high vs low) | 1.64 (1.02 - 2.65) | < 0.05 | * |
|  |  |  | Biopsy | CD68 count (high vs low) | 1.01 (1.01 - 1.01) | < 0.01 | ** |
|  | Teng | 2015 | Biopsy | MDSC count (high vs low) | 1.50 (0.64 - 3.53) | 0.34 |  |
|  | Alderdice | 2017 | Resection | CD56 expression (positive vs negative) | 0.28 (0.11 - 0.73) | < 0.01 | ** |
| Immune activity, checkpoints and exhaustion | Sato | 2014 | Resection | MHC class 1 expression (high vs low) | 0.22 (0.03 - 1.75) | 0.15 |  |
|  | Chen | 2019 | Biopsy | PD-L1 expression (high vs low) | 0.15 (0.05 - 0.47) | < 0.01 | ** |
|  |  |  | Resection | PD-L1 expression (high vs low) | 0.15 (0.05 - 0.47) | < 0.01 | ** |
|  | Boustani | 2020 | Biopsy | PD-L1+ cells (high vs low) | 0.60 (0.32 - 1.12) | 0.10 |  |
|  |  |  | Biopsy | PD-L1+ cells (third tertial vs first and second tertials) | 0.42 (0.23 - 0.79) | < 0.05 | * |
|  |  |  | Resection | PD-L1+ cells (high vs low) | 0.54 (0.29 - 1.02) | 0.05 |  |
|  |  |  | Biopsy - resection | Delta PD-L1 (>0.2 vs <0.2) | 0.96 (0.52 - 1.78) | 0.90 |  |
|  |  |  | Biopsy - resection | PD-L1 trajectory (high - high vs low - low) | 0.33 (0.13 - 0.86) | 0.07 |  |
|  |  |  | Biopsy - resection | PD-L1 trajectory (high - low vs low - low) | 1.15 (0.51 - 2.59) | 0.07 |  |
|  |  |  | Biopsy - resection | PD-L1 trajectory (low - high vs low - low) | 1.01 (0.45 - 2.25) | 0.07 |  |
|  | Chiang | 2019 | Biopsy | PD-L1 expression (high vs low) | 0.39 (0.14 - 1.08) | 0.07 |  |
|  |  |  | Resection | PD-L1 expression (high vs low) | 0.16 (0.05 - 0.48) | < 0.01 | ** |
|  | Shao | 2017 | Resection | tumour PD-L1 expression (high vs low) | 2.02 (0.45 - 9.13) | 0.36 |  |
|  |  |  | Resection | immune-cell PD-L1 expression (high vs low) | 1.48 (0.52 - 4.23) | 0.46 |  |
|  | Saigusa | 2016 | Resection | semiquantitative PD-L1 expression (high vs low) | 2.28 (1.03 - 5.02) | < 0.05 | * |
|  | Lim | 2017 | Biopsy - resection | PD-L1 trajectory (high - high vs low - low) | 0.09 (0.02 - 0.43) | < 0.01 | ** |
|  |  |  | Biopsy - resection | PD-L1 trajectory (high - low vs low - low) | 0.12 (0.03 - 0.54) | < 0.01 | ** |
|  |  |  | Biopsy - resection | PD-L1 trajectory (low - high vs low - low) | 0.83 (0.18 - 3.85) | 0.81 |  |
|  | Huang | 2021 | Resection | PD-L1 promoter cg15837913 methylation (high vs low) | 2.68 (1.18 - 6.08) | 0.06 |  |
|  |  |  | Resection | PD-L1 promoter cg19724470 methylation (high vs low) | 3.24 (1.03 - 10.20) | < 0.05 | * |
|  | Huang | 2018 | Biopsy | cytoplasmic HMGB1+ or PD-1+ vs both negative | 0.47 (0.10 - 2.17) | 0.33 |  |
|  | Teng | 2015 | Biopsy | CTLA-4 expression (high vs low) | 0.6 (0.26 - 1.42) | 0.25 |  |
|  | Schollbach | 2019 | Resection | IDO1 expression (high vs low) | 0.42 (0.11 - 1.59) | 0.20 |  |
|  | Chiang | 2019 | Biopsy | IFNγ and PD-L1 high vs either low | 0.54 (0.18 - 1.64) | 0.28 |  |
|  |  |  | Resection | IFNγ and PD-L1 high vs either low | 0.16 (0.05 - 0.56) | < 0.01 | ** |
|  | Peng | 2021 | Resection | PD-1 expression (high vs low) | 0.57 (0.12 - 2.57) | 0.46 |  |
|  |  |  | Resection | Tumour TIM-3 expression (high vs low) | 0.61 (0.20 - 1.84) | 0.38 |  |
|  |  |  | Resection | Immune-cell TIM-3 expression (high vs low) | 0.69 (0.23 - 2.05) | 0.50 |  |
|  |  |  | Resection | Tumour LAG-3 expression (high vs low) | 1.10 (0.38 - 3.21) | 0.87 |  |
|  |  |  | Resection | Immune-cell LAG-3 expression (high vs low) | 1.07 (0.36 - 3.17) | 0.90 |  |
| Other features | Chiang | 2021 | Germline | TIM3-R140L genotype (TT vs GG or GT) | 1.62 (0.64 - 5.41) | 0.34 |  |
|  |  |  | Germline | P2RX7-E496A genotype (GG vs AA or AG) | 1.22 (0.20 - 4.00) | 0.79 |  |
|  |  |  | Germline | TLR1-S602 genotype (TT vs GG or GT) | 1.84 (0.73 - 6.18) | 0.21 |  |
|  |  |  | Germline | FPR1-E346A genotype (CC vs AA or AC) | 2.41 (1.21 - 5.21) | < 0.05 | * |
